# The Japanese breast cancer society clinical practice guidelines for pathological diagnosis of breast cancer, 2022 edition

**DOI:** 10.1007/s12282-023-01518-6

**Published:** 2023-11-07

**Authors:** Naoko Honma, Masayuki Yoshida, Keiichi Kinowaki, Rie Horii, Yuka Katsurada, Yuya Murata, Ai Shimizu, Yuko Tanabe, Chikako Yamauchi, Yutaka Yamamoto, Hiroji Iwata, Shigehira Saji

**Affiliations:** 1 Department of Pathology, Faculty of Medicine, Toho University, 5-21-16 Omori-Nishi, Ota-Ku, Tokyo, 143-8540 Japan; 2 Department of Diagnostic Pathology, National Cancer Center Hospital, 5-1-1 Tsukiji, Chuo-Ku, Tokyo, 104-0045 Japan; 3 Department of Pathology, Toranomon Hospital, 2-2-2 Toranomon, Minato-Ku, Tokyo, 105-8470 Japan; 4 Department of Pathology, Saitama Cancer Center, 780 Komuro, Ina, Kita-Adachi-Gun, Saitama, 362-0806 Japan; 5 Pathology and Laboratory Medicine, National Defense Medical College, 3-2, Namiki, Tokorozawa, Saitama, 359-8513 Japan; 6 Department of Pathology, NHO Tokyo Medical Center, 2-5-1, Higashigaoka, Meguro-Ku, Tokyo, 152-0021 Japan; 7 Department of Surgical Pathology, Hokkaido University Hospital, Kita-14, Nishi-5, Kita-Ku, Sapporo, 060-8648 Japan; 8 Department of Medical Oncology, Toranomon Hospital, 2-2-2 Toranomon, Minato-Ku, Tokyo, 105-8470 Japan; 9 Department of Radiation Oncology, Shiga General Hospital, 4-1-1 KyomachiShiga Prefecture, Otsu City, 520-8577 Japan; 10 Department of Breast and Endocrine Surgery, Kumamoto University Hospital, 1-1-1 Honjo, Chuo-Ku, Kumamoto, 860-8556 Japan; 11 Department of Breast Oncology, Aichi Cancer Center Hospital, 1-1 Kanokoden, Chikusa-Ku, Nagoya, 464-8681 Japan; 12 Department of Medical Oncology, Fukushima Medical University, 1 Hikarigaoka, Fukushima City, Fukushima 960-1295 Japan

**Keywords:** Breast cancer, Guideline, 2022, Pathological diagnosis

## Introduction

This article is an English digest of the guidelines for pathological diagnosis published in The Japanese Breast Cancer Society (JBCS) Clinical Practice Guidelines in Japanese by Kanehara & Co., Ltd. in July 2022. In the 2022 edition, six Reviews, eight Basic Questions (BQs), and six Future Research Questions (FRQs) were included. Unlike for other fields, no Clinical Question (CQ) was included because an interventional study is rarely performed in diagnostic pathology. BQs refer to routinely used pathological methods supported by extensive evidence. The BQs for human epidermal growth factor receptor 2 (HER2) and hormone receptors have been revised according to revised international guidelines. FRQs refer to relatively new areas or non-routine methods supported by less evidence. Two new FRQs have been established for PD-L1 and cancer gene panel testing.

## Guidelines for pathological diagnosis

### Review 1: intrinsic subtype, molecular biological risk classification, and pathological diagnosis

Pathological substitution of ‘intrinsic subtype’ using the status of the estrogen receptor (ER), progesterone receptor (PgR), HER2 and Ki-67 is prevalent in clinical practice for breast cancer [1]. Definitions vary, but ‘luminal A-like’ is currently usually used for biologically indolent hormone receptor (ER and/or PgR)-positive and HER2-negative tumors, whereas ‘luminal B-like’ is used for biologically aggressive tumors. Pathological factors to differentiate between ‘luminal A-like’ and ‘luminal B-like’ include pathological grade, and expression levels of ER, PgR and Ki-67. A multi-gene expression assay (MGEA) is commonly used in Western countries to differentiate between these tumors [2], and one such MGEA, Oncotype DX, was approved for pharmaceutical use in August 2021 and is expected to be widely used in Japan. However, the definitions of ‘luminal A-like’ and ‘luminal B-like’ are still unclear [3] and are used in conventional, conceptual and varying ways. The meaning of these terms needs to be shared among medical staff and should be noted in published articles.

Triple-negative (negative for ER/PgR/HER2) breast cancer (TNBC) can be molecularly classified as basal-like, luminal-AR (androgen receptor), immune-activated, and claudin-low. Pathological classification of TNBC using EGFR, CK5/6, AR, cell-adhesion molecules, and tumor infiltrating lymphocytes (TIL) has been suggested to optimize treatment of TNBC. In Japan, health insurance has been permitted since 2019 for treatment with immune checkpoint inhibitors (ICIs) and for the designated immunohistochemical PD-L1 examination for each ICI (companion diagnostics). The relationship between molecular and pathological information requires updating based on the rapid development of research, diagnosis, and treatment of breast cancer.

### Review 2: appropriate pre-analytical methods required for pathological materials

Formalin-fixed and paraffin-embedded (FFPE) tissue are used for both routine pathological examination and for immunohistochemistry (IHC), *in-situ* hybridization (ISH), MGEAs, or cancer gene panel examinations. The pre-analytical steps, which are particularly important to assure the quality of the protein, mRNA or DNA, include the procedures of formalin fixation, paraffin embedding, and preparation of sliced specimens [4, 5].

1. Formalin fixation: materials from patients should be fixed with abundant formalin (about ten times the volume of the material) as soon as possible. The American Society of Clinical Oncology/College of American Pathologists (ASCO/CAP) HER2 guideline recommends fixation with 10% buffered formalin within one hour. Leaving materials without fixation for more than 30 min at room temperature should be avoided. If immediate fixation is difficult, materials should be preserved at 4℃ and fixed within 3 h. For a large specimen, separate fixation of tumorous tissue or making a cut near the tumor is effective for prompt tumor fixation. For the formalin fixation time, the ASCO/CAP HER2 guideline recommends 6–72 h. For genomic examination, fixation with 10% buffered formalin within 48 h is recommended.
2. FFPE tissue and preparation of sections: for IHC, 4-μm thick FFPE sections are recommended. The ASCO/CAP HER2 guideline recommends staining within 6 weeks after section preparation. For genomic examination, use of FFPE tissue blocks within 3 years after preparation is desirable.
3. Quality control: in the last decade, use of 10% buffered formalin has increased from < 50% to > 70% of sites in Japan due to recognition of the importance of fixation for companion diagnostics or genomic examination. Results from FFPE materials are strongly affected by the pre-analytical process. Thus, recognition of the importance of this process and internal or external quality control is important for maintaining the quality of the protein or nucleic acid in the pathological material. External quality control for hormone receptors and HER2 was performed in 2019 by the Japan Pathology Quality Assurance System.

### Review 3: pathological grading of invasive ductal carcinoma

There has been a focus on pathological grade, in addition to lymph node status, as a factor that affects clinical outcomes. The usefulness of pathological (histological or nuclear) grade has particularly been emphasized in invasive ductal carcinoma, and pathological grade is an independent prognostic factor. The Nottingham histological grading system is the most widely used for pathological grading worldwide [6] and comprises three scores for tubule formation, nuclear atypia, and mitoses. Nuclear grade, which includes scores for nuclear atypia and mitoses, is used for invasive ductal carcinoma in the JBCS General Rules for Clinical and Pathological Recording of Breast Cancer [7]. The histological and nuclear grades are both divided into three grades: patients with grade 1 (low-grade) tumors have favorable outcomes, whereas those with grade 2/3 (moderate/high-grade) tumors have poor outcomes [6, 7]. However, subjectivity and low reproducibility of pathological grading are problematic. Training for histological or nuclear grading may be useful to increase the interobserver concordance rate.

### Review 4: lesions for which benign/malignant discrimination is difficult using fine needle aspiration cytology or needle biopsy

Fine needle aspiration cytology (FNA), core needle biopsy (CNB), and vacuum-assisted biopsy (VAB) are recommended for pathological diagnosis of breast lesions due to lower invasiveness compared with surgical biopsy and high diagnostic accuracy (BQ1). However, differences between FNA/CNB/VAB results and examinations of surgically resected material are relatively common due to sampling errors by clinicians, missing of lesions by pathologists, observational difficulties caused by artifacts, and difficulty of diagnosis with cytology or minute tissue samples. Lesions that are difficult to diagnose in cytology or needle biopsy include benign lesions such as intraductal papilloma, ductal adenoma, fibroadenoma, mastopathy, radial sclerosing lesions, and malignant lesions such as low-grade ductal carcinoma in situ (DCIS) or classic lobular carcinoma in-situ (LCIS).

The terms ‘flat epithelial atypia (FEA)’, ‘atypical ductal hyperplasia (ADH)’, and ‘atypical lobular hyperplasia (ALH)’ have been used for atypical lesions that do not meet the criteria for carcinoma. The definition of these lesions and the risk of occurrence of breast carcinoma in a person with each lesion is described in detail in the WHO 5th edition [8]. Briefly, FEA is a columnar cell lesion composed of low-grade atypical cells and is viewed as a precursor lesion of ADH or low-grade DCIS, but the risk of FEA alone (without ADH/ALH) for invasive breast cancer is low. ADH is defined as a lesion composed of uniform low-grade atypical cells that do not meet the criteria for carcinoma in quantity or quality. The relative risk of ADH for invasive breast cancer is 3–5 times higher than normal, and 10–20% of ADH in biopsy material may be upgraded to DCIS in surgical material [8]. The diagnostic concordance rate of ADH is relatively low (40–60%), but may be increased by immunohistochemical examination [8]. ALH is a proliferative lesion of low-grade atypical cells in a terminal duct-lobular unit. The discriminating criteria between ALH and LCIS are not standardized, and the term ‘lobular neoplasia’ includes both lesions. The relative risk of occurrence of breast cancer after diagnosis of ALH is reportedly 4–5 times higher than normal [8].

FEA, ADH and ALH are originally defined using surgical material. Lesions diagnosed as such in cytology or needle biopsy include both definitive lesions and a wide spectrum of lesions from benign to malignant, causing a lower diagnostic concordance rate [9]. The next step for such lesions should be selected among surgical biopsy, re-needle biopsy, or follow-up, based on the clinical and pathological findings. In the JBCS General Rules for Clinical and Pathological Recording of Breast Cancer, the following procedure is recommended for reporting the pathological diagnosis: 1) whether the material is inadequate or adequate; 2) if the material is adequate, whether the lesion is normal/benign, indeterminate, a suspected malignancy, or malignant; and 3) detailed information on the likely histological diagnosis.

### Review 5: utility of immunohistochemistry (IHC) for benign/malignant discrimination in intraductal epithelial proliferative lesions

IHC is increasingly used for pathological diagnosis of intraductal epithelial proliferative lesions to differentiate between a benign and malignant status. Usual ductal hyperplasia (UDH) exhibits a mosaic staining pattern for high molecular weight cytokeratin (HMW-CK) such as CK5/6 or CK14, whereas ADH/low-grade DCIS is negative [10, 11]. UDH exhibits a scattered staining pattern for ER, whereas ADH/low-grade DCIS is diffusely and strongly positive for ER [12]. IHC using CK5/6, CK14, and ER is useful for discrimination of intraductal epithelial proliferative lesions [13]; however, some pitfalls should be noted: 1) a pure benign papillary lesion is often negative for HMW-CK; 2) apocrine lesions are negative for HMW-CK and ER irrespective of their benign/malignant status, and IHC should not be used for discrimination of this status for apocrine lesions; and 3) high-grade DCIS, which is easily diagnosed as malignant, is frequently HMW-CK-positive and ER-negative. Thus, IHC is useful, but should be appropriately used in combination with hematoxylin–eosin slides to increase the accuracy and concordance rate of pathological diagnosis for the atypical epithelial proliferative lesions.

### Review 6: confirming the diagnosis of a lesion suspected to be metastatic breast cancer

IHC may be useful to confirm the diagnosis for a lesion suspected to be metastatic breast cancer.

- (1)CK7 and CK20: A combination of CK7 and CK20 can be used to identify a primary lesion of metastatic adenocarcinoma. Breast cancer is largely CK7-positive/CK20-negative. In a review, Tot et al. found the following CK7/CK20 rates in patients with breast cancer: CK7-positive/CK20-negative, 88%; CK7-negative/CK20-positive, 1%; CK7-positive/CK20-positive, 11% [14]^.^
- (2)Biomarkers suggesting breast origin: ER/PgR are frequently expressed in primary lesions of breast cancer; however, their sensitivity and specificity at metastatic sites are not high enough to confirm a breast origin by themselves. GCDFP-15/mammaglobin/GATA3 are also frequently expressed in breast cancer; however, the positive rate differs among studies. GCDFP-15/mammaglobin/GATA3 are also commonly expressed in tumors of skin appendages or salivary glands [15].
- (3)Specific biomarkers for tumors with an origin other than the breast: Positive results for specific biomarkers may be useful to exclude a breast origin (e.g. TTF-1/napsin A for pulmonary adenocarcinoma, TTF-1/thyroglobulin for thyroid cancer, CDX2 for colorectal cancer, uroplakin-III for urothelial cancer, PAX8 for ovarian/endometrial cancer); however, it should be noted that a few breast cancers may give positive results for these markers [16].

IHC using the biomarkers described above is useful for diagnosis of a lesion suspected to be metastatic breast cancer; however, each biomarker can produce atypical results, suggesting the need for comprehensive analyses and panel diagnosis using multiple markers.

## BQ1: what is recommended first as a diagnostic procedure for a breast lesion: fine needle aspiration cytology (FNAC), core needle biopsy (CNB), or vacuum-assisted biopsy (VAB)?

### 1. For a non-palpable lesion, CNB or VAB is recommended first due to diagnostic accuracy.

### 2. For a lesion clinically suspected to be benign, FNAC is recommended first due to cost-effectiveness and relatively high diagnostic accuracy.

### 3. For a lesion clinically suspected to be malignant, CNB or VAB is recommended first due to diagnostic accuracy and probable abundant pathological information, such as histological type or biomarker presentation.

FNAC is inexpensive and its diagnostic accuracy is high for a palpable lesion, but low for a non-palpable lesion [17, 18].

## BQ2: is pathological examination recommended to evaluate the effect of neoadjuvant therapy?

### Pathological examination is recommended to evaluate the effect of neoadjuvant therapy and to predict the outcome; however, methods or criteria for examination are not standardized, which may cause discordant results.

In the General Rules for Clinical and Pathological Recording of Breast Cancer 18^th^ edition, the pathological effect of neoadjuvant therapy for invasive lesions is classified into grade 0 (no response), grade 1a (mild response, less than 1/3), grade 1b (moderate response, 1/3–2/3), grade 2a (high grade changes, 2/3 or more), grade 2b (extremely high grade, a few remaining cancer cells), and grade 3 (no invasive cancer cells remaining). These criteria have been reported to be useful to predict outcomes and to have a moderate diagnostic concordance rate [19, 20].

Pathologic complete response (pCR) after neoadjuvant chemotherapy may be a predictor of a favorable outcome in HER2-positive cancer or TNBC [21, 22]; however, the definition of pCR differs among studies. The absence of both an invasive lesion and a nodal metastatic lesion is generally required for a diagnosis of pCR, but there is no consensus for an in-situ lesion.

In reporting of the pathological effect of neoadjuvant therapy, details should be clearly stated regarding the examined area and the evaluation criteria and results, including the presence or absence of invasive/in-situ/nodal lesions or evidence of tumor disappearance.

## BQ3: why and how are hormone receptors examined?

### The hormone receptor status should be examined immunohistochemically to determine the indication for endocrine therapy

Immunohistochemical examination of ER and PgR is strongly recommended for all primary breast cancers. IHC should be performed using pharmaceutically approved primary antibodies according to each manufacturer’s instruction. Nuclear staining is quantified as a percentage (e.g., J-score) or as a combination of a percentage and intensity (e.g., Allred score) [23, 24]. Cases with < 1% positive cells for both ER and PgR are classified as hormone receptor-negative and excluded from endocrine therapy. Cases with ≥ 1% positive cells for ER or PgR are classified as hormone receptor-positive and indicated for endocrine therapy; however, for cases with low ER/PgR expression (e.g. < 10%), endocrine therapy should be considered carefully based on the risk/benefit balance because of its relatively poorer effect in such tumors compared with those with high ER/PgR expression [4]. It is recommended that the pathologist reports the percentage of positively stained tumor cells (with intensity information if the Allred score is used), and that the clinician selects medications based on a comprehensive consideration of all the clinicopathological information.

## BQ4: why and how is HER2 examined?

### To determine the indication for anti-HER2 therapy, the HER2 status should be examined by immunohistochemistry (IHC) or in situ hybridization (ISH)

HER2 status is a predictor for anti-HER2 therapy and clinical outcome, and should be examined for the invasive component of breast cancer. In Japan, IHC is usually performed first and results in a score of 0, 1 + , 2 + , or 3 + , where 0/1 + is HER2-negative, 3 + is HER2-positive, and 2 + is equivocal for HER2. In 2 + cases, ISH should be performed to evaluate HER2 gene amplification. The procedure should follow the algorithm shown in the 2018 ASCO/CAP HER2 guideline [5]. Re-examination of HER2 should be performed if the HER2 result contradicts other pathological findings [5]. Determination of low HER2 expression is described in detail in another article on this topic published in *Breast Cancer* [25].

## BQ5: How should the surgical margin be determined pathologically in partial mastectomy?

### 1. The pathological method to determine the surgical margin of partially removed breast material is not standardized. The pathological report should include the absence or presence of tumors, the method used for examination, and the details of the nearest tumor to the margin (type of tumor element, amount, etc.).

### 2. Intraoperative margin examination may not lead to an accurate diagnosis.

The following details of the nearest tumor to the margin should be reported: shortest distance from the margin, type of tumor element (invasive/in-situ, histology, grade, presence/absence of comedo necrosis), and amount [26, 27]. The pathological accuracy of intraoperative margin examination is high enough to be recommended; however, indefinite lesions, sampling errors, or artifacts may cause an incorrect diagnosis.

## BQ6: how should a sentinel lymph node be examined pathologically?

### 1. A sentinel lymph node should be examined pathologically using hematoxylin–eosin sections. One-step nucleic acid amplification (OSNA) can substitute for the usual pathological examination.

### 2. Routine immunohistochemical examination is not recommended for pathological diagnosis of a sentinel lymph node.

A metastatic lesion can be classified into isolated tumor cells (ITC, ≤ 0.2 mm in diameter or ≤ 200 tumor cells), micrometastasis (≤ 2 mm), and macrometastasis (> 2 mm) based on the largest dimension of the metastatic lesion. To detect macrometastasis, examination of slices at every 2-mm interval is required. Nodes with ITC should not be counted as positive nodes.

Health insurance can be used for OSNA, as a molecular examination of metastasis in sentinel lymph nodes [28]. OSNA can reduce the workload of pathologists and technicians. Metastasis that is only detected immunohistochemically has less clinical importance [29].

## BQ7: is evaluation of nuclear grade or comedo necrosis recommended for DCIS?

### Nuclear grading of tumor cells or evaluation of comedo necrosis has become increasingly important for selection of cases to be treated with partial mastectomy without radiation or to be followed up without treatment; however, the evaluation methods are not standardized and the interobserver consistency rate is not sufficiently high.

The pathological grade of DCIS affects the clinical outcome [30]. There are several nuclear grading systems that to date are not standardized. The Van Nuys classification incorporates the presence or absence of comedo necrosis, as well as the nuclear grade. [8, 31]. An incorrect understanding of the definition of comedo necrosis may cause discordance in its evaluation.

## BQ8: is examination of the hormone receptor or HER2 status recommended for needle biopsy material?

### 1. Examination of the hormone receptor or HER2 status in needle biopsy specimens is required to determine the neoadjuvant treatment strategy.

### 2. Examination of the hormone receptor or HER2 status in needle biopsy specimens is not necessarily required for cases for which neoadjuvant therapy is not planned.

In the examination of hormone receptors and HER2, needle biopsy specimens can be substituted for surgical specimens. For cases without neoadjuvant treatment, hormone receptors can be reexamined in needle biopsy specimens if they are negative in surgical specimens. For cases with neoadjuvant treatment, the status of hormone receptors or HER2 may be reexamined in the surgical specimens to predict the clinical outcome [32].

## FRQ1: which kind of invasive breast cancer should be examined for Ki-67? How should Ki-67 be estimated?

### 1. Ki-67 is useful to predict the clinical outcome of ER-positive/HER2-negative breast cancer, but not for selection of medication; thus, treatment should not be determined using Ki-67 alone.

### 2. The method for evaluation of Ki-67 has not been standardized.

Detection of Ki-67 nuclear staining is considered to be positive staining regardless of the intensity. Distribution of the Ki-67 labeling index (LI) differs among subtypes. Setting a cut-off value is difficult, but indexes of < 5% and ≥ 30% are largely accepted as low and high cut-offs, respectively. A Ki-67 labeling index of 10–25% should not be used as an indication for adjuvant therapy [3, 33].

## FRQ2: is examination of hormone receptors or HER2 recommended for DCIS?

### 1. Hormone receptors may be examined in DCIS if adjuvant endocrine therapy is being considered.

### 2. To date, there is no evidence to recommend HER2 examination in DCIS.

Neither hormone receptor nor HER2 status in DCIS predict ipsilateral breast tumor recurrence with invasion after breast conserving therapy. If adjuvant endocrine therapy is being considered to reduce DCIS recurrence in the conserved breast or events in the contralateral breast, hormone receptors may be examined in DCIS [4].

## FRQ3: is examination of cell block material from metastatic breast cancer recommended to determine the treatment strategy?

### For a distant metastasis, a cell block from aspiration material or body cavity fluid is likely to be available for biomarker examination using IHC or ISH; however, health insurance is currently not applicable for biomarker examination using a cell block from metastatic breast cancer.

A FFPE cell block can be used for IHC or ISH, and the results of ER/PgR/HER2 in a cell block sample are comparable with those from a corresponding tissue sample [34]. A cell block sample can also be used to confirm breast origin by IHC using GATA3, GCDFP-15, or mammoglobin [35].

## FRQ4: is evaluation of tumor infiltrating lymphocytes (TIL) useful in invasive ductal carcinoma?

### A TIL-high status is a favorable prognostic factor for some breast cancers and may also be a predictive marker for neoadjuvant chemotherapy. TIL evaluation is now being standardized; however, diagnostic reproducibility should be verified. Thus, TIL may be evaluated, but this is currently not necessary.

TIL is a prognostic factor for TNBC treated with adjuvant chemotherapy [36]. Evidence is accumulating that TIL is also a predictive factor for neoadjuvant therapy in TNBC or HER2-positive tumors, but this is still insufficient to support use of TIL to determine the treatment strategy [3]. TIL may be evaluated using the criteria suggested by the International TILs Working Group [37] but the criteria are still in the trial stage.

## FRQ5: how should PD-L1 be examined in invasive breast cancer?

### PD-L1 examination in invasive breast cancer should be performed immunohistochemically using the companion diagnostic (CDx) for each target immune checkpoint inhibitor (ICI)

Two ICIs, atezolizumab and pembrolizumab, are approved for unresectable/recurrent PD-L1-positive TNBC in Japan. To determine the indication for each ICI, PD-L1 should be examined immunohistochemically using the designated CDx: Ventana OptiView PD-L1 (SP142)® for atezolizumab and PD-L1 IHC 22C3 pharmDx Dako® (Code No. SK006) for pembrolizumab [38, 39]. Staining and evaluation procedures are strictly regulated for each CDx. The result of each CDx cannot be substituted for the result of the other CDx [40]. Training for this procedure is recommended to increase the diagnostic reproducibility of PD-L1 examination.

## FRQ6: what kind of cancer gene panel testing is available and what kind of material is needed for the test?

### OncoGuide™ NCC Oncopanel System and FoundationOne® CDx have been approved in Japan (October, 2021) for cancer gene panel testing using pathological material. For accurate molecular diagnosis, quality management in the preanalytical step is most important.

Slices from FFPE tissue are required for these tests. FFPE samples collected within 3 years, fixed for 6–48 h with 10% neutral buffered formalin solution, and containing tumor cells that are ≥ 30% of all nucleated cells are desirable to assure the quality of nucleic acids [41].
